# Pediatric bipolar disorder: Preliminary results of a retrospective study using a nationwide administrative database

**DOI:** 10.1192/j.eurpsy.2021.538

**Published:** 2021-08-13

**Authors:** A. Campos, M. Gonçalves-Pinho, A.R. Ferreira, A. Freitas, L. Fernandes

**Affiliations:** 1 Faculty Of Medicine, University of Porto, Porto, Portugal; 2 Cintesis – Center For Health Technology And Services Research, Faculty of Medicine, University of Porto, Porto, Portugal; 3 Department Of Psychiatry And Mental Health, Centro Hospitalar do Tâmega e Sousa, Penafiel, Portugal, Penafiel, Portugal; 4 Department Of Community, Information And Health Decision Sciences (medcids), Faculty of Medicine, University of Porto, Porto, Portugal; 5 Psychiatry Service, Centro Hospitalar Universitário de São João, Porto, Portugal

**Keywords:** bipolar disorder, Hospital admissions, adolescents, epidemiology

## Abstract

**Introduction:**

Bipolar disorder (BD) is a severe and chronic illness characterized by episodic changes in mood. The average onset of BD symptoms is estimated between 18 and 22 years. However, many adults retrospectively report symptoms onset in childhood or adolescence. Over the last decades, pediatric bipolar disease (PBD) has been the focus of increased attention mainly due to controversies surrounding its prevalence, diagnosis and treatment in the pediatric population.

**Objectives:**

To analyze pediatric hospitalizations related to BD held in mainland Portuguese public hospitals between 2000 and 2015.

**Methods:**

This retrospective observational study analyzed all pediatric (<18 years old) inpatient episodes from 2000 to 2015 with a primary BD diagnosis, using an anonymized administrative database including all hospitalization from mainland Portuguese public hospitals. ICD-9-CM codes 296.x were used (excluding codes 296.2x; .3x and .9x). Age at admission, admission type and date, sex, charges and length of stay (LoS) were analyzed.

**Results:**

A total of 348 hospitalizations were analyzed from 258 patients. Patients were mainly young girls (60.6%), with a mean age of 15.24±1.87 years. The majority of the admissions were urgent (81.0%), and the median LoS was 14 days (IQR: 7; 24). Mean hospitalization charges were 3503.1€ with a total sum of 1.2M€ for all the episodes.

**Conclusions:**

PBD hospitalizations occur predominantly in female patients during adolescence. The majority of them are urgent admissions. Descriptive studies will help to describe and characterize sociodemographic and clinical trends in PBD in order to better prevent acute hospitalizations with inevitable social and economic implications.

